# Chloromethane‐Enabled Quaternization of Linear Polyglycerol Amines and Their Application as Antibacterial Agents

**DOI:** 10.1002/marc.202500111

**Published:** 2025-04-18

**Authors:** Natalie Hanheiser, Merlin Kleoff, Katharina Achazi, Abhishek Singh, Sebastian Riedel, Rainer Haag

**Affiliations:** ^1^ Freie Universität Berlin Takustraße 3 14195 Berlin Germany; ^2^ Freie Universität Berlin Fabeckstraße 34/36 14195 Berlin Germany

**Keywords:** alkylation, amines, antibacterial effects, biological activity, disinfectants, gas‐phase reactions, polymer

## Abstract

In this study, the safe and scalable *N*‐methylation of primary amines in a linear polyglycerol (LPG) backbone structure is reported with altering molecular weight using gaseous chloromethane for the generation of quaternary ammonium groups. All polymers are subsequently analyzed for their antibacterial and antibiofilm properties against drug‐resistant *Staphylococcus aureus* (*S. aureus*), showing that the implementation of quaternary ammonium groups to a polymer backbone structure is an efficient way to generate new antimicrobial agents. Thereby, the molecular weight of the polymer backbone structure strongly correlates to its antibacterial effect and can be altered depending on the desired application.

## Graphical abstract

This study details a scalable process for synthesizing quaternary ammonium compounds (QACs) from linear polyglycerols using gaseous chloromethane. The resulting compounds demonstrate effective antibacterial and antibiofilm properties against drug‐resistant *S. aureus*, highlighting a promising approach in developing antimicrobial agents from cheap, less hazardous chemicals.

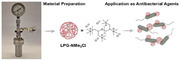

## 1 Introduction

*N*‐ and *O*‐alkylation reactions are mandatory for the synthesis of many pharmaceuticals,^[^ ^1^ ^]^ including antibiotics, for instance, Clarithromycin,^[^ ^2^ ^]^ Chloramphenicol,^[^ ^3^ ^]^ and Fluoroquinolones.^[^ ^4^ ^]^ In addition, QACs are essential to generate effective infection control as they are commonly used disinfectants and antiseptics.^[^ ^5^ ^]^ Their common structure consists of one central nitrogen atom (head) to which four alkyl or aryl chains (tail) are attached. The mechanism of their biocidal action is based on the adherence of the alkylammonium cation on the bacterial cell surface and their diffusion through the cell wall. This damage to the cell wall results in a release of inner cell compartments and finally, cell death.^[^ ^5b^ ^]^ In recent years, frequent reports on the increasing resistance of certain microorganisms, like *S. aureus*, toward QACs have been reported.^[^ ^6^ ^]^

**Figure 1:**
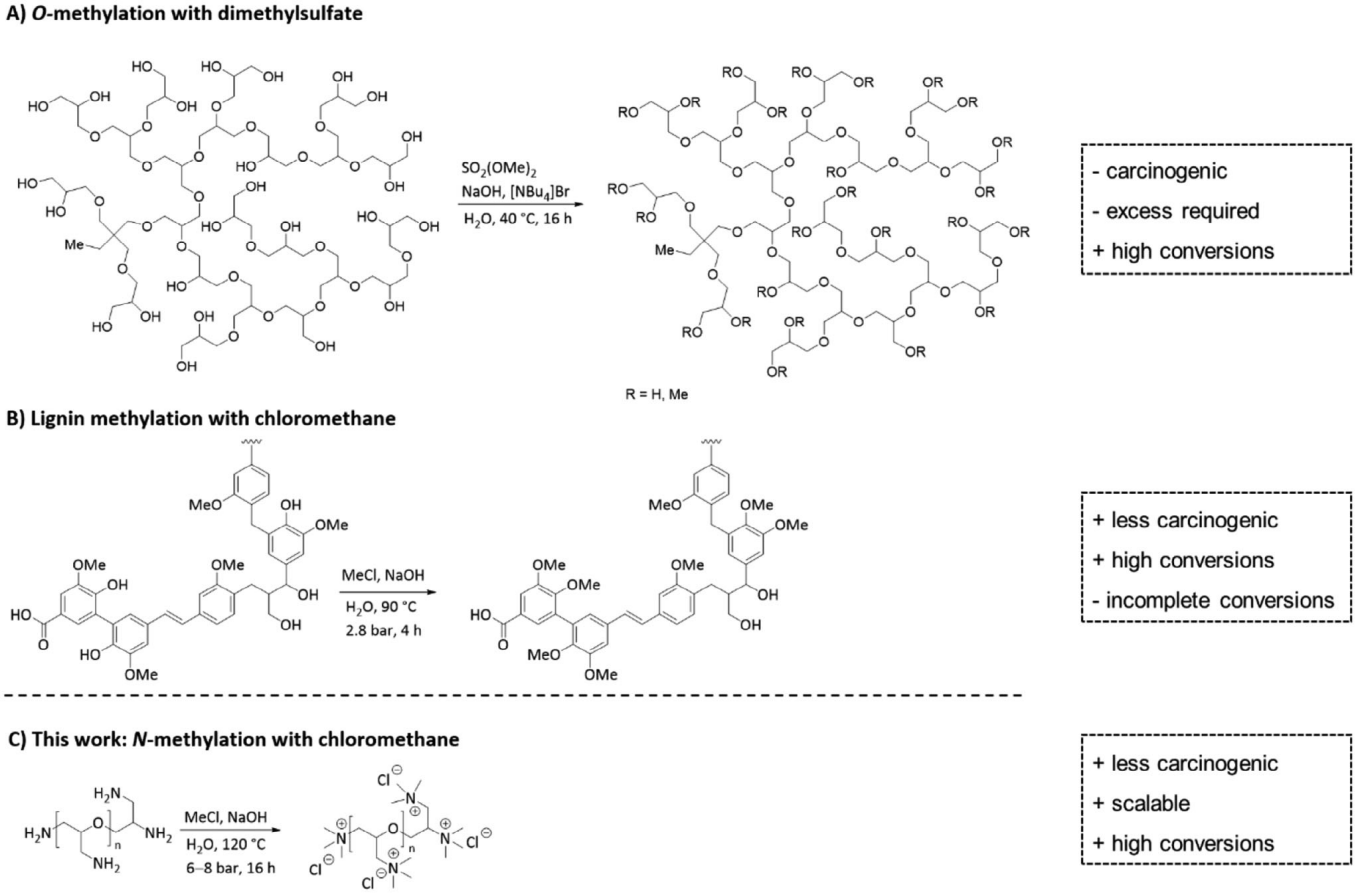
O‐ and N‐methylation of different polymer backbone structures. A) *O*‐methylation of hyperbranched polyglycerol using dimethylsulfate following the synthetic procedure of Koschek and co‐workers.^[^ ^12^ ^]^ B) *O*‐methylation of lignin using gaseous chloromethane following the synthetic procedure of Gagné and co‐workers.^[^ ^18^ ^]^ C) *N*‐methylation of linear polyglycerol using gaseous chloromethane (proposed within this work).

One approach for the generation of new QACs lies in the incorporation of the quaternary ammonium group into a multivalent polymer backbone structure.^[^ ^7^ ^]^ The usage of polyglycerols for the polymer backbone structure is highly attractive; based on their structure, polyglycerols can be seen as the multifunctional analog of poly(ethylene glycol).^[^ ^8^ ^]^ In comparison to ethylene glycol, glycerol is a “green” starting material that is mainly obtained from the vegetable oil industry. Polyglycerols are water‐soluble, biocompatible, and open for post‐modifications over several hydroxy groups, which makes them attractive for biomedical and pharmaceutical applications.^[^ ^9^ ^]^ The synthesis of QACs often relies on the use of cancerogenic alkylation agents such as dimethyl sulfate, diethyl sulfate,^[^ ^10^ ^]^ or methyl iodide.^[^ ^11^ ^]^ As described by Koschek and co‐workers, the successful *O*‐methylation of hyperbranched polyglycerols was achieved using an excess of dimethyl sulfate (**Figure** **1**A).^[^ ^12^ ^]^ Efforts were made to replace these cancerogenic alkylation agents with the less toxic dimethyl carbonate and methanol, as they are considered to be more environmentally friendly. Unfortunately, they typically yield only low degrees of quaternization.^[^ ^13^ ^]^ In comparison to that, the use of chloromethane (methyl chloride, CH_3_Cl) is highly attractive. Chloromethane is a methylation agent that is widely used in industrial chemistry for the manufacture of silicone^[^ ^14^ ^]^ and methylcellulose^[^ ^15^ ^]^ as well as for the synthesis of surfactants,^[^ ^16^ ^]^ and pharmaceuticals.^[^ ^17^ ^]^ Recently, chloromethane was used for the *O‐*alkylation of softwood and hardwood kraft lignin's (Figure 1B).^[^ ^18^ ^]^ With the aid of chloromethane, which appears as a gas at standard conditions (0 °C, 1 atm), the contamination of the environment with liquid cancerogenic methylation agents can be avoided, which leads to improved working‐safety.^[^ ^18^ ^]^ The use of gaseous chloromethane provides a full quaternization of all functional groups without the need to perform further halogen exchange reactions. On top of that, the cheap costs and the improved atom economy of chloromethane allow it to be implemented at a large scale.

Until now, the functionalization of LPG using gaseous chloromethane as a methylation agent for the generation of an antibacterial quaternary ammonium group remains unknown. Within our research, we present the full quaternization of different molecular weight (M_W_) LPG using chloromethane and their application as antibacterial agents to selectively overcome emerging problems related to growing bacterial resistance while achieving a sustainable as well as highly efficient synthesis (Figure 1C).

## 2 Results and Discussion

**Figure 2:**
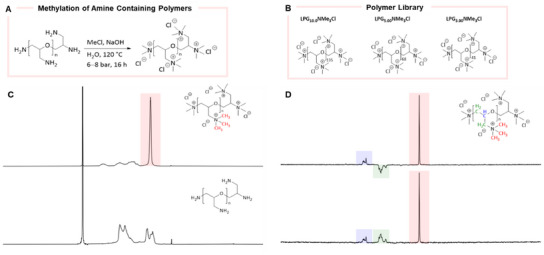
A) Schematic overview of the *N*‐methylation of all primary amines to the corresponding quaternary amines in a linear polyglycerol (LPG) backbone structure. B) Library of different‐sized LPGs functionalized with chloromethane. C) ^1^H NMR spectra of LPG with a quaternary ammonium group (top) and LPG amine (bottom). D) DEPT ^13^C NMR (top) and IG ^13^C NMR (bottom) spectra of LPG with a quaternary ammonium group.

Our aim was to develop a chloromethane‐based methylation procedure for the quaternization of LPG that would be both environmentally friendly and relatively cost‐effective to be implemented at a large scale. With a solubility of 6.52 g kg^−1^ at 30 °C and 1  bar, chloromethane is moderately soluble in water. The solubility of chloromethane in aqueous solution can be increased by applying pressure to the system, which results in a biphasic liquid–liquid system.^[^ ^18^ ^]^ The hydroxy groups of polyglycerol can be easily converted by mesylation, azidation, and subsequent Staudinger reduction into primary amines, as previously reported.^[^ ^19^ ^]^ To take up the hydrogen chloride, which is formed as a by‐product during the methylation of amines with chloromethane, the pH value of the reaction mixture needs to be adjusted to pH 10 and above (**Figure** **2**A). Unfortunately, in aqueous alkali solution, chloromethane hydrolyses to methanol as a competing reaction. Consequently, it was needed to determine conditions where the rate of methylation exceeds that of chloromethane hydrolysis. After a rapid screening of different reaction conditions, we found that the reaction proceeds best using 3.30 equiv CH_3_Cl per primary amine of 1.00 equiv LPG (Table S2, Supporting Information). Employing the optimized conditions, LPG was dissolved in 20.0 mL water per 1.00 g LPG, and the pH value of the solution was adjusted to pH 14 using sodium hydroxide (4.00 equiv per primary amine of 1.00 equiv LPG). The solution was placed in an 80.0 mL stainless steel autoclave, and chloromethane (3.30 equiv per primary amine of 1.00 equiv LPG) was condensed in. The reaction mixture was heated to 120 °C for 16 h, reaching an initial pressure of 8.00 bar, which drops over the course of the reaction due to the consumption of chloromethane. Notably, the reaction was conducted on a one‐gram scale.

The successful conversion of all primary amines into the corresponding quaternary ammonium group was proven by ^1^H NMR spectroscopy in which the peak of the methyl‐groups appears as a singlet at 3.26 ppm (Figure 2C; Figures S1–S6, Supporting Information). The full conversion of all primary amines into the corresponding quaternary ammonium group was confirmed by calculating the ratio between the multiplet of the polymer backbone structure and the singlet of the protons at the methyl group of the quaternary ammonium functionality. Using IG ^13^C NMR and DEPT ^13^C NMR spectroscopy, the methyl carbon appears as a singlet at 54.2 ppm. Whereas the methylene carbon appears as a multiplet at 65.9–68.2 ppm and the methine carbon appears as a multiplet at 72.6–73.5 ppm (Figure 2D; Figures S10 and S11, Supporting Information).

To evaluate the scope of the developed method and demonstrate its efficacy, linear polyglycerol amines (LPG‐NH₂) of varying molecular weights were selected and subjected to methylation reactions. The chosen polyglycerol backbones included LPG_10.0_ (M_W_ = 10.0 kDa, n = 135), LPG_5.00_ (M_W_ = 5.00 kDa, n = 68), and LPG_3.00_ (M_W_ = 3.00 kDa, n = 41). Despite differences in the number of amine groups present in each LPG variant, characterization (Figures S1–S6, Supporting Information) confirmed complete conversion to the methylated products in all cases, highlighting the robustness and efficiency of the developed approach.

We were specifically interested in understanding the antibacterial effect of our polymers against drug‐resistant *S. aureus* as they are known to contain the *qacC* gene that causes resistance toward QACs and ethidium bromide.^[^ ^6^, ^20^ ^]^

All three polymers were tested against MRSA and *Escherichia coli* (*E.coli*) at different inhibitor concentrations.

**Figure 3:**
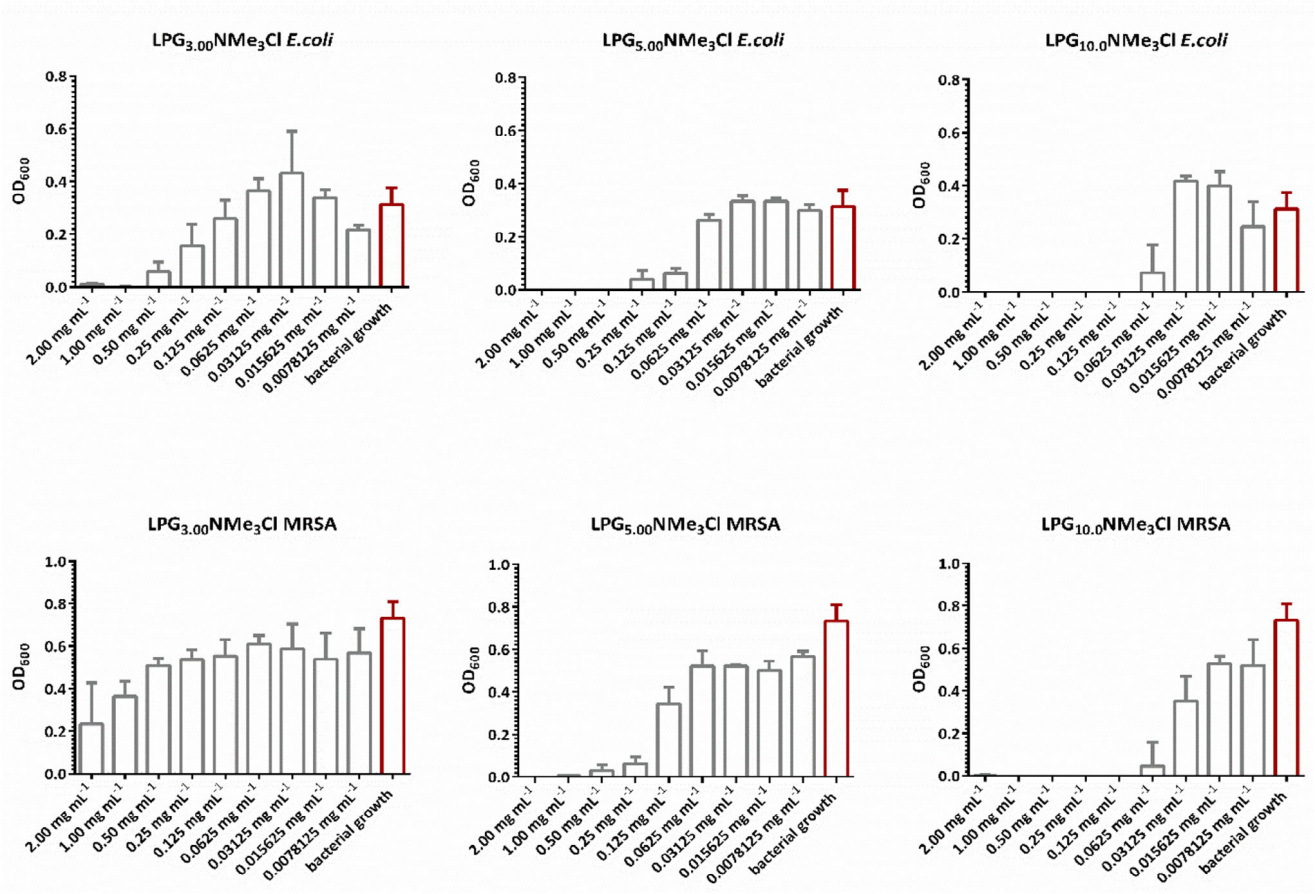
Antibacterial effect of different polymer inhibitors at different concentrations (mg mL^−1^) on *E. coli* and methicillin‐resistant *S. aureus* bacterial growth assessed via measurements of the optical density of the bacteria‐containing media at 600 nm (OD_600_) (mean ± SD, n  =  3).

**Figure** **3** shows the effect of different inhibitor concentrations on the growth of the bacteria in the medium, which is accessed by the optical density of the bacteria‐containing media at 600 nm (OD_600_). We found that the inhibition efficiency of the LPG derivatives with a quaternary ammonium group increases with a higher molecular weight of the polymer backbone structure. In the case of the 3.00 kDa polymer, the bacterial growth of *E. coli* increases again at an inhibitor concentration of 0.125 mg mL^−1^. The same phenomenon can be observed for the 5.00 and 10.0 kDa polymer at an inhibitor concentration of 0.0625 and 0.03125 mg mL^−1^. In comparison to *E. coli*, the 3.00 kDa polymer system shows no antibacterial effect against MRSA. In the case of the 5.00 and 10.0 kDa polymer, an increase in the bacterial growth occurs at inhibitor concentrations of 0.125 and 0.03125 mg mL^−1^. Taken together, we observe an increase in the antibacterial properties of the polymers with an increased molecular weight. This observation can be explained by the higher amount of quaternary groups that correlate to the higher molecular weight of the polymer. For example, the 3.00 kDa polymer has ≈41 quaternary ammonium groups, while the 5.00 and 10.0 kDa polymers have ≈68 and 135 quaternary ammonium groups. An increased amount of quaternary ammonium groups leads to an increased positive charge density of the polymer (Table S1, Supporting Information). Due to the higher density of positive charge, the electrostatic interactions between the polymer and the bacteria are increased, resulting in an increased adherence of the polymer to the bacteria and, thereby, to a higher inhibition efficiency.^[^ ^21^ ^]^ Overall, the polymers show a stronger inhibition effect against *E. coli* than against drug‐resistant MRSA. On one hand, this observation can be explained by the higher pathogenicity of MRSA in comparison to *E. coli*. On the other hand, the cell wall of Gram‐negative *E*. *coli* has an overall thinner peptidoglycan layer than that one of Gram‐positive MRSA, which might be the reason why a higher amount of quaternary ammonium groups is needed to effectively destroy the cell wall of Gram‐positive MRSA.^[^ ^22^ ^]^

Besides genetically acquired resistance, bacterial resistance can be obtained physically by the formation of a biofilm.^[^ ^23^ ^]^ Encouraged by the antibacterial efficiency of the quaternary ammonium group containing polyglycerol derivatives, we were interested in testing those compounds for their potential to eradicate already existing biofilm on surfaces. Therefore, MRSA in suspension was seeded in a well plate at a concentration of 10^6^ CFU mL^−1^, and the mature biofilm was grown over 72 h incubation at 37 °C. Afterward, all three polymers were added to the mature biofilm at different concentrations for another 24 h, followed by crystal violet staining. All three polymers were tested at a very high concentration above and at their corresponding minimum inhibitory concentration (MIC) value.

**Figure 4:**
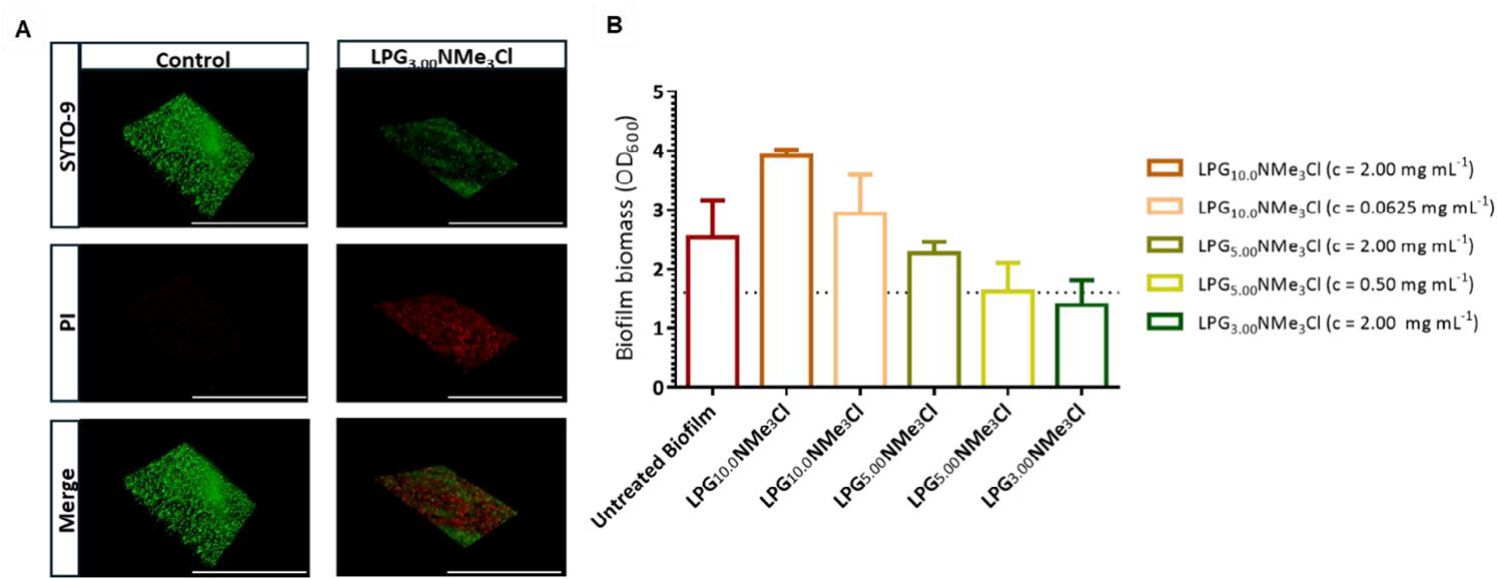
A) Confocal laser scanning microscopy images of MRSA mature biofilm showing the untreated control and LPG_3.00_ NMe_3_Cl treated biofilm (scale bar corresponds to 1 000 000 µm). B) Optical density (OD) at 600 nm in a.u. of the biofilm biomass of MRSA against different polymer derivatives at different concentrations (mg mL^−1^) (mean ± SD, n  =  3).

**Figure** **4**B illustrates the OD_600_ of the biofilm biomass against different polymer derivatives at varying concentrations. As shown in Figure 4B, only the polymer with the lowest molecular weight showed an effective biofilm biomass reduction of 50 % at a concentration of 2.00 mg mL^−1^. Whereas both polymer derivatives with a higher molecular weight of 5.00 and 10.0 kDa showed no biofilm‐eradicating properties (Figure 4B). This high increase in the biofilm eradication activity of LPG_3.00_NMe_3_Cl in comparison to the higher molecular weight derivatives might be due to its more effective diffusion in the biofilm; the two higher molecular weight derivatives can be trapped by the biofilms extracellular polymeric substances (EPS), which consist of crosslinked polysaccharides and proteins, thereby preventing the successful eradication of the biofilm.^[^ ^24^ ^]^

To confirm the potential of LPG_3.00_NMe_3_Cl to remove already existing biofilms from surfaces, Syto9 and Propidium iodide as Live/Dead staining and confocal laser scanning microscopy images were used. Here, a clear difference in the biofilm viability between the untreated and treated biofilm is visible (Figure 4A).

## 3 Conclusion

In summary, we developed a scalable and safe method for the alkylation of primary amines in a polyglycerol backbone structure using gaseous chloromethane. In comparison to other alkylation reagents, the use of chloromethane is highly efficient as it yields a full conversion of all primary amines to the corresponding quaternary ammonium group while being cheap and offering a good atom economy. On top of that, the contamination of the environment by liquid and carcinogenic alkylation agents can be prevented as gaseous chloromethane can simply be released from the reaction mixture. The successful methylation of LPG‐NH₂ across different molecular weights demonstrates the versatility and robustness of this method and provides a solid basis for extending its application beyond linear polyglycerols. The complete transformations observed in LPGs highlight the potential of the method for the modification of various amine‐containing macromolecules and pave the way for tailored surface functionalities in drug delivery, biomaterials, and nanomedicine. The obtained quaternary ammonium derivatives of LPG were further investigated regarding their antimicrobial potential. Interestingly, we found that the 10.0 kDa polymer derivate, due to its higher charge density, shows a higher antibacterial effect when it comes to inhibiting the bacterial growth of both *E. coli* and MRSA in solution. In contrast to that, the smaller M_w_ polymer derivate is more suitable for the eradication of already existing MRSA biofilms as it can effectively enter the biofilm matrix. In accordance with already existing literature, our results strongly emphasize that the incorporation of a quaternary ammonium group into a multivalent polymer backbone structure is an efficient approach to overcome bacterial resistance toward quaternary ammonium compounds. We anticipate that this method is generally applicable for the safe and scalable quaternization of different amine‐containing polymer backbone structures, enabling rapid access to new QACs with improved antibacterial properties.

## 4 Experimental Section

### Materials

All solvents and reagents were purchased from commercial suppliers, including: VWR International; Sigma–Aldrich, Carl Roth, Fisher Scientific, and Merck (Germany).

### Methods—Synthetic Procedure

The amine containing LPG derivatives was synthesized following the previously reported literature.^[^ ^19^ ^]^ For the preparation of LPG_n_‐NMe_3_Cl (with n = 3.00, 5.00, and 10.0 kDa), LPG_n_‐NH_2_ (n = 3.00, 5.00, 10.0 kDa) was dissolved in water in an 80.0 mL stainless steel autoclave. The pH value of the solution was set to pH 14 by using NaOH (4.00 equiv per NH_2_). The mixture was frozen with liquid nitrogen and degassed using the freeze‐pump‐thaw technique. Chloromethane (3.30 equiv per NH_2_) was condensed, and the reaction mixture was heated up to 120 °C, reaching an initial pressure of 8.00 bar. The reaction mixture was stirred for 16 h at this temperature while the pressure dropped to 6.00 bar due to the consumption of chloromethane. The obtained polymers were purified by dialysis for three days in water.

1H NMR (400 MHz, D2O, δ): 3.73 (m, 5H), 3.28 (s, 9H) ppm.

IG 13C NMR (600 MHz, D2O, δ): 73.0 (m, 1C), 68.9 (m, 2C), 54.3 (s, 3C) ppm.

DEPT 13C NMR (600 MHz, D2O, δ): 73.0 (m, 1C), 68.9 (m, 2C), 54.3 (s, 3C) ppm.

### Methods—In Vitro Microdilution Assay

The bacteria strain *Escherichia coli* DH5α (obtained from Invitrogen) and methicillin‐resistant *Staphylococcus aureus* (DMSZ No. 13661)^[^ ^25^ ^]^ were streaked out on an LB agar‐plate and incubated overnight at 37 °C. The next day, one colony was picked and diluted in 5.00 mL LB‐medium. The bacteria solution (10^6^ CFU mL^−1^) was transferred to a 96‐well plate, and the different compounds at different concentrations were added to the bacteria. The bacteria and the compound were incubated for 24 h at 37 °C. The optical density at 600 nm was measured before and after the incubation and compared to each other to access the bacterial growth in the bacteria in the solution.

### Methods—In Vitro Biofilm Eradication Assay

Methicillin‐resistant S. aureus was streaked out on an LB agar‐plate and incubated overnight at 37 °C. The next day, one colony was picked and diluted in 5.00 mL LB‐medium. The bacteria in solution (10^6^ CFU mL^−1^) were seeded to a 24‐well plate (2.00 mL well^−1^) and incubated for 72 h. After three days, the mature biofilms were washed with PBS (3 × 2.00 mL well^−1^), and the biofilm was incubated for another 24 h at 37 °C together with the compound at different concentrations in LB‐medium. The next day, the biofilms were washed with PBS (3 × 2.00 mL well^−1^). Then, 0.1% crystal violet was added to the wells (400 µL well^−1^) for 20 min. Afterward, the biofilm was washed with PBS (1 × 2.00 mL well^−1^), and ethanol was added to the biofilm (2.00 mL well^−1^). The Biofilm Biomass was determined by measuring the Optical density at 600 nm using the Epoch 2 plate reader.

### Methods—In Vitro Biofilm Viability Assay

The Biofilm Viability was accessed by confocal laser scanning microscopy. Therefore, methicillin‐resistant *S. aureus* (10^8^ CFU mL^−1^) was seeded in an eight‐well ibidi slide (200 µL well^−1^) and cultured for 72 h at 37 °C to get the mature biofilm. Afterward, the different compounds were added to each well (200 µL well^−1^) and incubated for 4 h. Then, the Bacterial Viability Kit Live/Dead (BacLight, ThermoFisher Scientific) was used to distinguish between live and dead bacteria cells. Confocal images were taken by using an inverted confocal laser scanning microscope Leica DMI6000CSB SP8 (Leica, Wetzlar, Germany) using the manufacturer‐given LAS X software. The images were analyzed by ImageJ using the Fiji software.

## Conflict of Interest

The authors declare no conflict of interest.

## Supporting information



Supporting Information

## Data Availability

The data that support the findings of this study are available in the supplementary material of this article.
