# Supplementary material for: Chloromethane‐Enabled Quaternization of Linear Polyglycerol Amines and Their Application as Antibacterial Agents
Source: Macromol Rapid Commun. 2025 Apr 18;46(15):2500111. doi: 10.1002/marc.202500111 (PMC12344478; doi:10.1002/marc.202500111)
Supplement: Supplementary file 1 — Supporting Information [file MARC-46-2500111-s001.docx]

Supporting Information

Chloromethane Enabled Quaternization of Linear Polyglycerol Amines and their Application as Antibacterial Agents

Natalie Hanheiser,^a^ Merlin Kleoff,^b^ Katharina Achazi,^a^ Abhishek Kumar Singh,^a^ Sebastian Riedel,^b^ and Rainer Haag*^a^

Table S1. Zeta potential measurement

| Compound | ζ -- potential [mV] | # of quaternary ammonium groups |
| --- | --- | --- |
| LPG_10.0_NMe_3_Cl | 55.0 | 135.0 |
| LPG_5.00_NMe_3_Cl | 33.2 | 68.0 |
| LPG_3.00_NMe_3_Cl | 30.3 | 41.0 |

Table S2. Screening of different reaction conditions

| Batch # | M_W_(Polymer) [kDa] | m(Polymer) [g] | equiv MeCl per NH_2_ group | DF [%] |
| --- | --- | --- | --- | --- |
| Batch 01 | 10.0 kDa | 0.05 | 3.3 | 94 |
| Batch 02 | 10.0 kDa | 0.10 | 3.3 | 94 |
| Batch 03 | 10.0 kDa | 0.10 | 3.3 | 93 |
| Batch 04 | 10.0 kDa | 1.00 | 3.3 | 93 |
| Batch 05 | 3.00 kDa | 1.00 | 3.3 | 87 |
| Batch 06 | 5.00 kDa | 1.00 | 3.3 | 93 |

Image deleted. Please check.

Figure S1. ^1^H NMR spectra of LPG_10.0_NMe_3_Cl (Batch 01).

Image deleted. Please check.

Figure S2. ^1^H NMR spectra of LPG_10.0_NMe_3_Cl (Batch 02).

Image deleted. Please check.

Figure S3. ^1^H NMR spectra of LPG_10.0_NMe_3_Cl (Batch 03).

Image deleted. Please check.

Figure S4. ^1^H NMR spectra of LPG_10.0_NMe_3_Cl (Batch 04).

Image deleted. Please check.

Figure S5. ^1^H NMR spectra of LPG_3.00_NMe_3_Cl (Batch 05).

Image deleted. Please check.

Figure S6. ^1^H NMR spectra of LPG_5.00_NMe_3_Cl (Batch 06).

Image deleted. Please check.

Figure S7. ^1^H NMR spectra of LPG_3.00_NH_2_.

Image deleted. Please check.

Figure S8. ^1^H NMR spectra of LPG_5.00_NH_2_.

Image deleted. Please check.

Figure S9. ^1^H NMR spectra of LPG_10.0_NH_2_.

Image deleted. Please check.

Figure S10. DEPT ^13^C spectra of LPG_10.0_NMe_3_Cl (Batch 04).

Image deleted. Please check.

Figure S11. IG ^13^C spectra of LPG_10.0_NMe_3_Cl (Batch 04).
